# High genotype diversity and zoonotic potential of *Enterocytozoon bieneusi* in yaks (*Bos grunniens*) from Ganzi Tibetan Autonomous Prefecture, Sichuan Province[Fn FN1]

**DOI:** 10.1051/parasite/2023044

**Published:** 2023-09-27

**Authors:** Xin Yang, Ying-Ying Fan, Dan-Jiao Yang, Shuang Huang, Jun-Wei Wang, Xu Chen, Min Zhang, Yi-Wen Liu, Qiang Li, Jun-Ke Song, Guang-Hui Zhao

**Affiliations:** 1 College of Veterinary Medicine, Northwest A&F University Yangling 712100 China; 2 Animal Husbandry Science Institute of Ganzi Tibetan Autonomous Prefecture Kangding 626000 China; 3 College of Veterinary Medicine, Huazhong Agricultural University Wuhan 430070 China; 4 Engineering Research Center of Efficient New Vaccines for Animals, Ministry of Education Yangling 712100 China; 5 Key Laboratory of Ruminant Disease Prevention and Control (West), Ministry of Agriculture and Rural Affairs Yangling 712100 China; 6 Engineering Research Center of Efficient New Vaccines for Animals, Universities of Shaanxi Province Yangling 712100 China

**Keywords:** *Enterocytozoon bieneusi*, Yak, Genotype, Zoonotic potential

## Abstract

*Enterocytozoon bieneusi* is a common pathogen in humans and various animals, threatening the breeding industry and public health. However, there is limited information on the molecular characteristics of *E. bieneusi* in yaks, an economically important animal mainly domesticated in the Qinghai Tibet Plateau in China. In the present study, nested PCR targeting the ITS gene region was applied to investigate the positive rates and genetic diversity of *E. bieneusi* in 223 faecal samples of yaks from three locations in Ganzi Tibetan Autonomous Prefecture, Sichuan Province. The total positive rate of *E. bieneusi* was 23.8% (53/223). Significant differences in positive rates were identified among yaks from three locations (χ^2 ^= 8.535, *p* = 0.014) and four age groups (χ^2^ = 17.259, *p* = 0.001), with the highest positive rates in yaks from Yajiang and aged < 6 months, respectively. Sequence analysis identified seven known (EbpC, LW1, LQ10, PigEBITS5, ESH-01, J and BEB4) and five novel (Ganzi1–5) ITS genotypes. Phylogenetic analysis showed eight genotypes (EbpC, LW1, LQ10, PigEBITS5, ESH-01, Ganzi1, Ganzi2 and Ganzi4) in group 1 and three genotypes (J, BEB4 and Ganzi3) in group 2, indicating high genotype diversity and zoonotic potential of *E. bieneusi* in yaks from Ganzi. Considering the increasing zoonotic genotypes in yaks in the present study compared with previous findings, interventions should be developed to reduce the potential transmission of *E. bieneusi* between humans and animals.

## Abstract (fr)

*Enterocytozoon bieneusi* est un agent pathogène courant chez l'homme et chez divers animaux, menaçant l'industrie de l'élevage et la santé publique. Cependant, il existe peu d'informations sur les caractéristiques moléculaires d’*E. bieneusi* chez les yaks, un animal important pour l’économie, principalement domestiqué sur le plateau du Qinghai au Tibet en Chine. Dans la présente étude, une PCR imbriquée ciblant la région du gène ITS a été appliquée pour étudier la positivité et la diversité génétique d’*E. bieneusi* dans 223 échantillons fécaux de yaks provenant de trois sites de la préfecture autonome tibétaine de Ganzi, province du Sichuan. Le taux total de positivité pour *E. bieneusi* était de 23,8 % (53/223). Des différences significatives dans les taux positifs ont été identifiées parmi les yaks de trois emplacements (χ^2^ = 8,535, *P* = 0,014) et de quatre groupes d'âge (χ^2^ = 17,259, *P* = 0,001), avec les taux positifs les plus élevés respectivement chez les yaks de Yajiang et ceux âgés de moins de 6 mois. L'analyse de séquence a identifié sept génotypes ITS connus (EbpC, LW1, LQ10, PigEBITS5, ESH-01, J et BEB4) et cinq nouveaux (Ganzi1–5). L’analyse phylogénétique a montré huit génotypes (EbpC, LW1, LQ10, PigEBITS5, ESH-01, Ganzi1, Ganzi2 et Ganzi4) dans le groupe 1 et trois génotypes (J, BEB4 et Ganzi3) dans le groupe 2, indiquant une diversité génotypique élevée et un potentiel zoonotique d’*E. bieneusi* chez les yaks de Ganzi. Compte tenu de l’augmentation des génotypes zoonotiques chez les yaks dans la présente étude par rapport aux résultats précédents, des interventions devraient être développées pour réduire la transmission potentielle d’*E. bieneusi* entre les humains et les animaux.

## Introduction

Microsporidia are important opportunistic pathogens that lead to significant economic losses in animal breeding worldwide [11, 41]. Among them, *Enterocytozoon bieneusi* is one of the most common zoonotic species and contributes to over 90% of human cases of microsporidiosis [30, 41]. Although *E. bieneusi* usually causes asymptomatic infection in both immunocompetent and immunodeficient individuals, it can also lead to gastrointestinal disorders, wasting and diarrhoea, especially for immunocompromised populations (e.g., HIV patients) and children [10, 24, 34, 62]. Meanwhile, infected hosts can release mature spores that contaminate water and food, threatening public health [38, 52]. Thus, the Environmental Protection Agency and the National Institutes of Health of the United States have listed *E. bieneusi* as a microbial contaminant candidate for waterborne transmission and a Class B biodefense pathogen, respctively [4].

Knowledge of the distribution and genetic characterisation of pathogens can shed new light on the prevention and control of diseases. Based on the molecular characterisation of the ITS gene locus of *E. bieneusi*, over 600 genotypes from 11 genetic groups (groups 1–11) with divergent host specificity have been recognised [20, 21, 61]. Within group 1, a few genotypes, such as D, EbpC, Type IV, Peru6, Peru8 and Peru11, have been widely reported in both humans and animals, reflecting significant zoonotic importance of genotypes in this group [20]. Genotypes in group 2 were previously reported to be specific in ruminants, but expanding of the host range for some genotypes (e.g., BEB4, BEB6, I and J) indicates potential zoonotic significance or cross-species transmission capabilities within this group [20, 48, 49, 60]. Host specificity of genotypes was commonly found in the groups 3–11, reflected by the unique existence of WL6 in rodents, PtEb VIII in cats, and CAF4 in humans and non-human primates [1, 16, 20, 25, 43]. Most genotypes within groups 3–11 showed limited zoonotic potential [20]. However, due to the limited genotypes reported in the groups 3–11, further studies on more samples from diverse hosts and geographical areas are needed to verify zoonotic potential of genotypes within these groups.

Yaks are a unique livestock resource distributed in the Qinghai Tibet Plateau and its adjacent high and subalpine areas. These animals can adapt to harsh environments such as very low temperatures, hypoxia, and extreme dryness, and are an important and unique livestock species in production [55]. Under grazing condition, yaks can be easily infected with various parasitic pathogens, and some zoonotic pathogens have been reported in yaks, such as *Cryptosporidium* spp. [5], *Echinococcus granulosus* [22] and *Toxoplasma gondii* [46], indicating zoonotic potential of those pathogens.

Previous studies have reported positive rates of *E. bieneusi* in yaks from Gansu, Qinghai, Tibet and Yunnan in China, with positive rates ranging from 1.1% to 7.2%, and 15 genotypes, including five zoonotic genotypes (BEB4, BEB6, I, J and D) and ten animal-adapted genotypes (CHN11, CHN12, CHN13, CHN14, CHC8, CAM2, COS-I, NESH5, WCY1 and YAK1) [26, 29, 51, 58, 59]. To further explore the distribution of *E. bieneusi* in yaks, the present study investigated the positive rates and genotype distribution of *E. bieneusi* in yaks from three main breeding areas in Ganzi Tibetan Autonomous Prefecture, and assessed the zoonotic potential of this pathogen in yaks.

## Materials and methods

### Ethics statement

This study was conducted under the approval and instructions of the ethics committee of Northwest A&F University (DY2022048).

### Sampling

**Figure 1:**
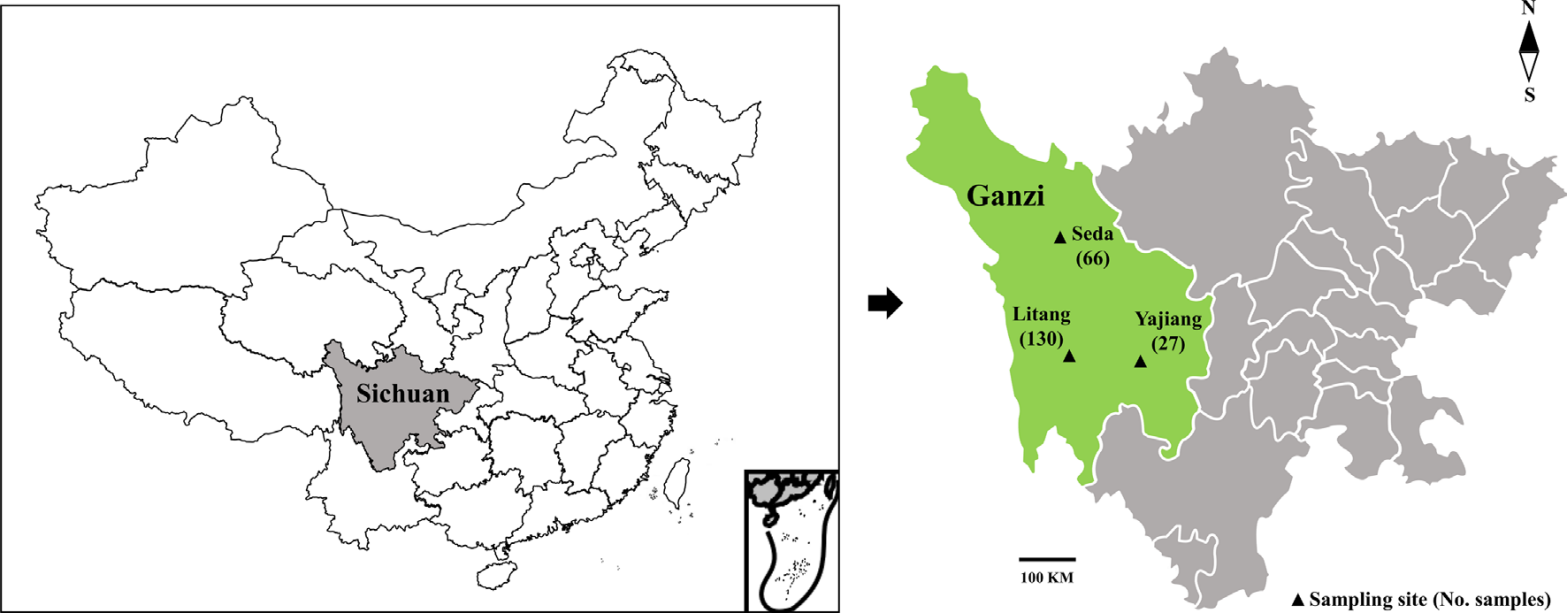
Geographical distribution of sampling sites in Ganzi Tibetan Autonomous Prefecture, Sichuan Province, China.

This study was conducted in Ganzi Tibetan Autonomous Prefecture, Sichuan Province, China. Ganzi is located in the west of Sichuan Province and the southeastern Tibetan plateau (97°22′–102°29′ E, 27°58′–34°20′ N), with 153,000 km^2^ and an average of over 4,000 m above sea level. As an important economic livestock species in Ganzi, yaks are usually raised in separate enclosures and seldomly have opportunities for contact with other animals. From October 2022 to April 2023, a total of 223 faecal samples were collected from yaks in Seda (*n* = 66), Litang (*n* = 130) and Yajiang (*n* = 27) in Ganzi (Fig. 1). For each analysed population, animals lived in enclosures, with about 10–20 animals sharing the same enclosure, and usually did not have close contact with humans except for farm owners. During the time of sampling, animals analysed in the present study did not show obvious symptoms, except for diarrhoea of 40 animals. All the samples were collected directly from the rectum of animals, placed in separated bags, marked with sample information (e.g., location, age and sex), transported to the parasitology laboratory of Northwest A&F University under cool conditions as soon as possible, and preserved in 2.5% potassium dichromate under 4 °C.

### Genomic DNA extraction

Faecal samples were washed in distilled water three times to remove potassium dichromate, and subsequently applied for total genomic DNA isolation using an E.Z.N.A. Stool DNA kit (Omega, Norcross, GA, USA), following the manufacturer’s instructions. The gDNA samples were stored at −20 °C.

### PCR amplification

The colonisation frequency of *E. bieneusi* was detected using nested PCR based on the ITS gene locus, and using the primers previously reported [43]. A nested PCR was conducted in a 25 µL reaction mixture containing 1× Rapid Taq Master Mix, 0.4 µM each primer, 1 µL gDNA for the primary PCR or 1 µL primary PCR product for the secondary PCR, under the following conditions for both rounds: an initial denaturing at 94 °C for 5 min, followed by 35 cycles of 94 °C for 45 s, 55 °C for 45 s and 68 °C for 1 min, and a final extension at 68 °C for 7 min. A positive control with gDNA isolated from *E. bieneusi*-positive samples preserved in our laboratory and a negative control with distilled water were included in each PCR reaction. Positive secondary PCR conducts will show a band of ~392 bp under a UV transilluminator after 1% agarose gel electrophoresis.

### Sequencing and sequence analysis

All positive secondary PCR products were sent to Sangon Biotech (Shanghai, China) for sequencing in both directions. The obtained sequences were identified to be *E. bieneusi* ITS gene by Blastn analysis at NCBI (https://blast.ncbi.nlm.nih.gov/Blast.cgi). To assess the relationship of *E. bieneusi* genotypes found in the present study, a phylogenetic tree was developed using the maximum-likelihood (ML) method with the General Time Reversible model and bootstrap evaluation of 1000 replicates within MEGA V6.0 [45].

### Statistical analysis

Differences in the positive rates of *E. bieneusi* in yaks among the location, age, sex and diarrhoea groups were analysed using a χ^2^ test in SPSS V18.0 (IBM, New York, NY, USA). Significant differences were confirmed if the *p-*value was less than 0.05.

### Nucleotide sequence accession numbers

Representative nucleotide sequences of *E. bieneusi* ITS gene in the present study have been submitted to GenBank under accession numbers OR023607–OR023620.

## Results

### Occurrence of *E. bieneusi* in yaks

Of the 223 faecal samples examined in the present study, 53 (23.8%) were positive for *E. bieneusi* in yaks based on the PCR-sequencing tool targeting the ITS gene locus (Table 1). There were significant differences among the positive rates of *E. bieneusi* in three locations (χ^2^ = 8.535; *p* = 0.014), with the highest in Yajiang (44.4%, 12/27), followed by Seda (25.8%, 17/66) and Litang (18.5%, 24/130). Meanwhile, a signiﬁcant difference in positive rates was also identiﬁed among four age groups (χ^2^ = 17.259; *p* = 0.001), with the highest in yaks aged <6 months (41.0%, 16/39), followed by 12–24 months (39.5%, 16/43), > 24 months (15.6%, 15/90) and 6–12 months (11.8%, 6/51). Although the positive rates of *E. bieneusi* varied among sex and diarrhoea groups, no significant differences were found (Table 1).

**Table 1:** Occurrence, genotypes and factors associated with *E. bieneusi* infection in yaks from Ganzi Tibetan Autonomous Prefecture.

Factor	No. examined	No. positive (%)	χ^2^	*p* value	Genotype (No.)
Location					
Yajiang	27	12 (44.4)	8.535	0.014	J (12)
Seda	66	17 (25.8)			EbpC (7), BEB4 (6), ESH-01 (1), LW1 (1), Ganzi1 (1), Ganzi2 (1)
Litang	130	24 (18.5)			BEB4 (18), PigEBITS5 (1), LQ10 (1), Ganzi3 (1), Ganzi4 (2), Ganzi5 (1)
Age (months)					
<6	39	16 (41.0)	17.259	0.001	J (11), BEB4 (3), Ganzi4 (2)
6–12	51	6 (11.8)			BEB4 (4), J (1), Ganzi5 (1)
12–24	43	16 (39.5)			BEB4 (11), EbpC (4), Ganzi1 (1)
>24	90	15 (15.6)			BEB4 (6), EbpC (3), ESH-01 (1), LW1 (1), PigEBITS5 (1), LQ10 (1), Ganzi2 (1), Ganzi3 (1)
Gender					
Male	62	12 (19.4)	0.149	0.699	BEB4 (11), Ganzi5 (1)
Female	60	10 (16.7)			BEB4 (6), PigEBITS5 (1), Ganzi3 (1), Ganzi4 (2)
NA	101	31 (30.7)			J (12), BEB4 (7), EbpC (7), ESH-01 (1), LW1 (1), LQ10 (1), Ganzi1 (1), Ganzi2 (1)
Diarrhoea					
Yes	40	7 (17.5)	1.057	0.304	BEB4 (4), EbpC (1), PigEBITS5 (1), Ganzi4 (1)
No	183	46 (25.1)			BEB4 (20), J (12), EbpC (6), ESH-01 (1), LW1 (1), LQ10 (1), Ganzi1-5 (1 per each)
Total	223	53 (23.8)			BEB4 (24), J (12), EbpC (7), ESH-01 (1), LW1 (1), PigEBITS5 (1), LQ10 (1), Ganzi1-3 (1 per each), Ganzi4 (2), Ganzi5 (1)

NA: not available.

### Distribution of *E. bieneusi* genotypes in yaks

Based on sequence analysis of the ITS gene locus of *E. bieneusi*, a total of 12 genotypes were identified in the 53 sequences in the present study, with seven known genotypes (BEB4, J, EbpC, LW1, LQ10, PigEBITS5 and ESH-01) and five novel genotypes (Ganzi1–5) (Table 1). No mixed infections of genotypes were identified in the present study. The novel genotypes identified in the present study have been re-sequenced, and the results indicated that these genotypes were truly novel. Ganzi1, Ganzi3 and Ganzi4 had three, one and two nucleotide substitutions compared with the genotypes EbpC (MN902235.1), BEB4 (MT231512.1) and CYG-1 (MZ479291.1), respectively. Ganzi2 had one nucleotide deletion compared with the genotype ESH-01 (KR902354.1). Ganzi5 had 31 nucleotide substitutions and two nucleotide deletions compared with the genotype XJH6 (MN704930.1). Among the identified genotypes, BEB4 was the commonest genotype found in 45.3% (24/53) of yak isolates, followed by J (22.6%, 12/53), EbpC (13.2%, 7/53), Ganzi4 (3.8%, 2/53) and other genotypes (1.9%, 1/53) (Table 1). There were sequence differences in genotype diversity among three locations, with six (BEB4, PigEBITS5, LQ10, Ganzi3–5), six (EbpC, BEB4, ESH-01, LW1, Ganzi1 and Ganzi2) and one (J) genotypes in Litang, Seda and Yajiang, respectively. Meanwhile, eight (BEB4, EbpC, ESH-01, LW1, PigEBITS5, LQ10, Ganzi2 and Ganzi3), three (J, BEB4 and Ganzi4), three (BEB4, J and Ganzi5) and three (BEB4, EbpC and Ganzi1) genotypes were identified in yaks aged >24 months, <6 months, 6–12 months and 12–24 months, respectively. More genotypes were found in female yaks (BEB4, PigEBITS5, Ganzi3 and Ganzi4) compared with male yaks (BEB4 and Ganzi5). A total of 11 genotypes were recognised in non-diarrhoea yaks (BEB4, J, EbpC, ESH-01, LW1, LQ10, Ganzi1–5), while only four were found in diarrhoeal yaks (BEB4, EbpC, PigEBITS5 and Ganzi4).

### Phylogenetic relationships of *E. bieneusi* genotypes

**Figure 2:**
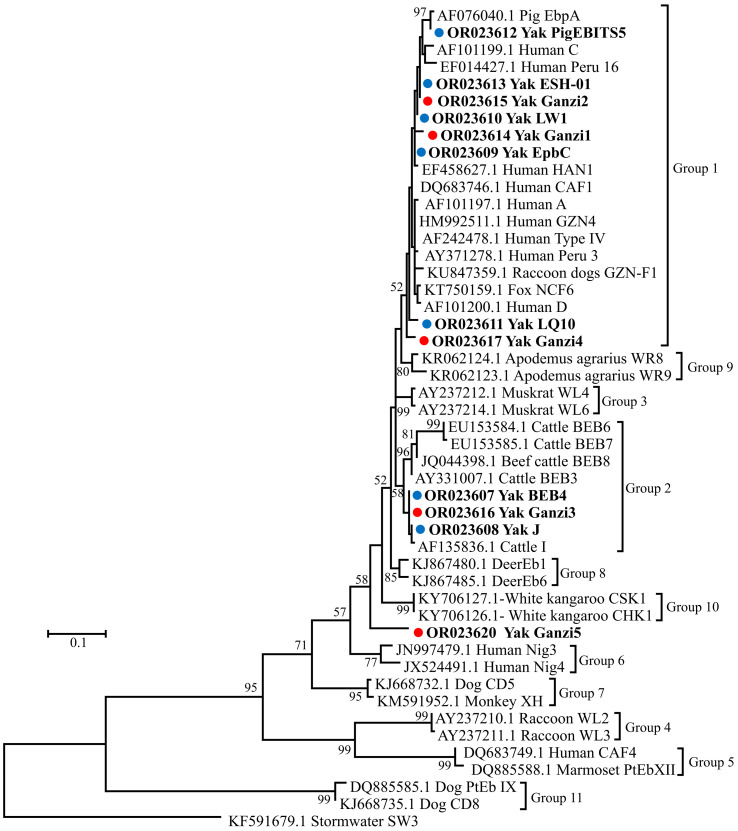
Phylogenetic relationships of *E. bieneusi* genotypes in this study, with reference sequences downloaded from GenBank based on the sequence analysis of the ITS locus using Maximum Likelihood analysis with the General Time Reversible model. Red and blue filled circles before the bold sample names represent novel and known genotypes identified in the present study, respectively. Bootstrap values (> 50) are indicated above the nodes. Scale bar indicates 0.1 nucleotide substitutions/site. Genotype SW3 from stormwater (KF591679.1) is used as the outgroup.

Phylogenetic analysis based on the ITS gene locus of *E. bieneusi* indicated eight genotypes (EbpC, LW1, LQ10, PigEBITS5, ESH-01, Ganzi1, Ganzi2 and Ganzi4) contributing to 28.3% (15/53) of the isolates, and belonged to the potentially zoonotic group 1 (Fig. 2). Meanwhile, three genotypes (J, BEB4 and Ganzi3) belonged to group 2, with increasing zoonotic potential, while genotype Ganzi5 did not belong to any known group (Fig. 2).

## Discussion

*Enterocytozoon bieneusi* is a common zoonotic pathogen threatening the health of humans and various animals [20]. Knowledge of the distribution and molecular genetics of pathogens could provide insights for the prevention and control of diseases. To further understand the colonisation frequency of *E. bieneusi* in bovine animals, the present study explored the occurrence and zoonotic potential of *E. bieneusi* in yaks from three locations in Ganzi Tibetan Autonomous Prefecture using PCR-sequencing targeted the ITS gene locus, and the results indicated high genetic diversity and zoonotic potential of *E. bieneusi* in yaks in this area.

Recently, *E. bieneusi* has been widely reported in bovine animals in China. In the present study, the positive rate of *E. bieneusi* in yaks was 23.8% (53/223), which was higher than that in yaks in Qinghai (7.0%; 7.2%) [26, 58], Gansu (1.13%) [29], and Tibet (5.0%) [51], water buffaloes in Jiangxi (5.6%) [19], Anhui (0.9%) [23] and Hunan (2.2%) [28], beef cattle in Jiangxi (3.9%) [19], Henan (5.4%) [28] and Shaanxi (19.7%) [50], and dairy cattle in most reported provinces in China, except for Heilongjiang (30.1%; 29.0%) [47, 63] and Jilin (37.6%) [60]. The disparities in positive rates of *E. bieneusi* in bovine animals were likely caused by discrepancies in animal species, host immune status, geographic regions, sampling sizes as well as management practices.

Notably, the infection of *E. bieneusi* in yaks was significantly related with age groups (Table 1). In this study, *E. bieneusi* was found in all age groups, with positive rates of 41.0% (16/39), 11.8% (6/51), 39.5% (16/43) and 15.6% (15/90) for yaks aged <6 months, 6–12 months, 12–24 months and >24 months, respectively, indicating lower positive rate in older yaks (>6 months) compared with younger yaks (<6 months). Similar results have also been reported in dairy cattle in Brazil and the Czech Republic [3, 13], indicating immunity to *E. bieneusi* in bovine animals likely increasing with the age. However, contrary results were found in a cross-sectional survey on the positive rates of *E. bieneusi* in dairy cattle on large farms across multiple states in the United States. Similarly, a longitudinal study of *E. bieneusi* on a dairy farm in Maryland, USA showed a higher positive rate of *E. bieneusi* in dairy cattle aged 7–24 months compared with animals aged under 6 months [6, 7, 37, 40].

Sequence analysis based on the ITS gene locus of 53 isolates in yaks found seven known genotypes and five novel genotypes, which could enrich our understanding of the genetic diversity of *E. bieneusi* in bovine animals. BEB4 was first found in cattle [44], and then reported in humans [34], pigs [60] and nonhuman primates [14]. Genotype J was not only reported in dairy cattle [17], but was also found in humans [60], nonhuman primates [56], donkeys [57], zebras, bears and meerkats [18], chickens [33] and wastewater treatment plants [53], indicating its wide host range and zoonotic potential. EbpC is also a genotype with zoonotic potential, and it has been reported in humans [42], nonhuman primates [54] and quite a few animals, such as pigs [8], sheep and cattle [12], dogs [15] and horses [32]. LQ10 was previously reported in *Marmota baibacina* (ON165748) in China, and the occurrence of this subtype in the present study enriched its host range. Genotype LW1 was first reported in lake water in China [54], and then identified in humans [49], swine [31], sheep [9] and deer [39], indicating the wide host range and zoonotic potential of this genotype. Meanwhile, genotype PigEBITS5 was first reported in swine in Massachusetts, USA [2], and then in humans [35], dogs [15], house mice [36] and raw wastewater [53], reflecting the broad host range and zoonotic potential of this genotype. Genotype ESH-01 has been reported in wastewater [27] and horses [32], and further studies are needed to explore its host range. Furthermore, five novel genotypes, namely Ganzi1–5, were first detected in the present study, and the host range and zoonotic potential of these genotypes will need to be evaluated in the future. Interestingly, all genotypes were J in yaks from Yajiang, while this genotype was absent in Seda and Litang, reflecting low genotypic diversity and unique genotypic distribution of yaks in Yajiang compared with the other two locations.

Further phylogenetic analysis indicated the occurrence of genotypes from both group 1 and group 2 with zoonotic potential in yaks in the present study, which is consistent with genotypes identified in yaks in Tibet [51, 58], Qinghai [26] and Gansu [29] and a cross-sectional survey across Qinghai, Yunnan and Tibet in China [59], reflecting zoonotic potential of these animals in the transmission of *E. bieneusi*. Two genotypes (CHN11 and CHN12) from group 1 and three genotypes (BEB4, I and J) from group 2 were identified in yaks in Qinghai [26]. Two genotypes (CHN14 and D) from group 1 and eight genotypes (I, J, BEB4, BEB6, COS-I, NESH5, CHC8 and CHN13) from group 2 were found in yaks in Tibet [51, 58]. WCY1 from group 1 and two genotypes (I and BEB4) from group 2 were reported in yaks in Gansu [29]. Yak1 from group 1 and BEB6 from group 2 were recognised in yaks in the cross-sectional survey across Qinghai, Yunnan and Tibet in China [59]. Compared with previous reports in yaks [26, 29, 51, 58, 59], the present study first identified the occurrence of ESH-01, PigEBITS5, LQ10, LW1, EpbC and three novel genotypes (Ganzi1, Ganzi2 and Ganzi4) from group 1 and novel Ganzi3 from group 2 in yaks, reflecting the possible expansion of genotypes in yaks. Among the five novel genotypes identified in yaks in this study, it is interesting to note that genotype Ganzi5 formed a separate branch in the phylogenetic tree, different from other genotypes with various host ranges within groups 1–11, indicating that Ganzi5 could be adapted to yaks, but more work needs to be done in the future to confirm this. Considering the increasing genotypes with zoonotic potential of *E. bieneusi* in yaks, interventions should be considered to prevent cross-transmission of *E. bieneusi* between humans and animals.

## Conclusions

In this study, we investigated *E. bieneusi* infection in yaks in Ganzi Tibetan Autonomous Prefecture, Sichuan Province, with a total positive rate of 23.8%, and significant differences were identified in the positive rates among location and age groups. A total of 12 genotypes, including seven known and five novel genotypes, were identified in yaks. Of these identified genotypes, eight and three genotypes were from the group 1 and group 2, respectively indicating potential importance and zoonotic potential of *E. bieneusi* in these yaks. These findings provide fundamental data for understanding transmission of *E. bieneusi* in yaks as well as other bovine animals.
